# Cx43 expression and function in the nervous system—implications for stem cell mediated regeneration

**DOI:** 10.3389/fphys.2014.00106

**Published:** 2014-03-18

**Authors:** Carola Meier, Katja Rosenkranz

**Affiliations:** ^1^Department of Anatomy and Cell Biology, Saarland UniversityHomburg/Saar, Germany; ^2^Department of Neuroanatomy and Molecular Brain Research, Ruhr University BochumBochum, Germany

**Keywords:** gap junction, stem cell, brain damage, hypoxia, transplantation

## Abstract

Pathological conditions of the brain such as ischemia cause major sensorimotor and cognitive impairments. In novel therapeutic approaches to brain injury, stem cells have been applied to ameliorate the pathological outcome. In several experimental models, including hypoxia-ischemia and trauma, transplantation of stem cells correlated with an improved functional and structural outcome. At the cellular level, brain insults also change gap junction physiology and expression, leading to altered intercellular communication. Differences in expression in response to brain injury have been detected in particular in Cx43, the major astrocytic gap junction protein, and its overexpression or deletion was associated with the pathophysiological outcome. We here focus on Cx43 changes in host tissue mediated by stem cells. Stem cell-induced changes in connexin expression, and consecutively in gap junction channel or hemichannel function, might play a part in altered cell interaction, intercellular communication, and neural cell survival, and thereby contribute to the beneficial effects of transplanted stem cells.

## Brain injury: therapeutic approaches using stem cells

The consequences of brain damage caused by hypoxia or trauma are often detrimental. As causative therapies are still limited, regenerative therapies using stem cells have been proposed. Different animal models have been used to investigate the effects of stem cell transplantation on the outcome after nervous system injury, including those of perinatal hypoxia-ischemia, transient, and permanent ischemia as well as traumatic brain injury. Many studies report beneficial effects of stem cell transplantation on the structural, behavioral and cognitive outcome (Xiong et al., 2009; Mankikar, 2010; Rosenkranz and Meier, 2011; English et al., 2013; Lemmens and Steinberg, 2013). It has been demonstrated that lesion-induced sensorimotor deficits were ameliorated upon transplantation of stem cells from various sources including umbilical cord blood mononuclear cells (reviewed by Rosenkranz and Meier, 2011), neural stem cells (English et al., 2013), and mesenchymal stem cells (van Velthoven et al., 2010; Donega et al., 2013). The mechanisms underlying the amelioration of symptoms might depend on the type of stem cell transplanted. In the case of multipotent stem cells, the hypothesis concerning the principle mechanism has recently shifted from neural replacement via differentiation of stem cells toward the idea that transplanted cells enhance the endogenous regenerative capacity of the brain. Several pathways com into consideration for these indirect effects, including immunomodulation (Rosenkranz et al., 2013; Zhang et al., 2013), the secretion of neuronal survival factors (Neuhoff et al., 2007; Drago et al., 2013), enhancement of angiogenesis (Taguchi et al., 2004; Rosenkranz et al., 2012) as well as a reduction of astrocyte activation and neuro-inflammation (Wasielewski et al., 2012). As many of these pathways might be related to the transfer of second messenger molecules or other intercellular signals, gap junction communication may provide the means for their propagation.

One appealing hypothesis, which is in line with the indirect effects of stem cells outlined above, is that transplanted cells influence gap junction expression in the host via paracrine factors. We therefore focus here on connexin 43 (Cx43) gap junction protein expression in the host tissue receiving the stem cell transplant, and view these findings in the context of a therapeutic application after brain injury.

## Gap junction changes in host tissue mediated by stem cell transplantation

As outlined above, it is becoming recognized that transplanted stem cells interact with endogenous cells of the host and that this action protects cells from secondary damage. To understand the complex effects of transplanted stem cells on gap junctions in a pathophysiological context, we would first like to outline the changes in connexin expression following brain damage using the example of Cx43.

Cx43 is the major astrocytic connexin and, as such, an important mediator of CNS injury (reviewed by Contreras et al., 2004; Nakase and Naus, 2004; Davidson et al., 2013). Significant changes in both spatial and temporal expression of Cx43 were observed following CNS injury: In a rodent model of transient global ischemia, Cx43 immunoreactivity increased in the CA1/2 pyramidal subfields of the hippocampus (Rami et al., 2001). A similar increase of Cx43 immunoreactivity in hippocampal and striatal areas was observed in rats with moderate striatal damage induced by bilateral carotid occlusion (Hossain et al., 1994). Interestingly, animals with severe ischemic damage displayed zones of reduced staining within areas of strong Cx43 immunostaining (Hossain et al., 1994). The effect of reduced Cx43 staining within the lesion center in combination with higher Cx43 staining in the surrounding tissue was also observed after neonatal hypoxic-ischemic brain injury (Wasielewski et al., 2012). In this model, quantification of Cx43 expression revealed a significant overall increase of mRNA and protein levels upon injury (Wasielewski et al., 2012). This effect was most prominent 2 days after lesion.

**Figure 1:**
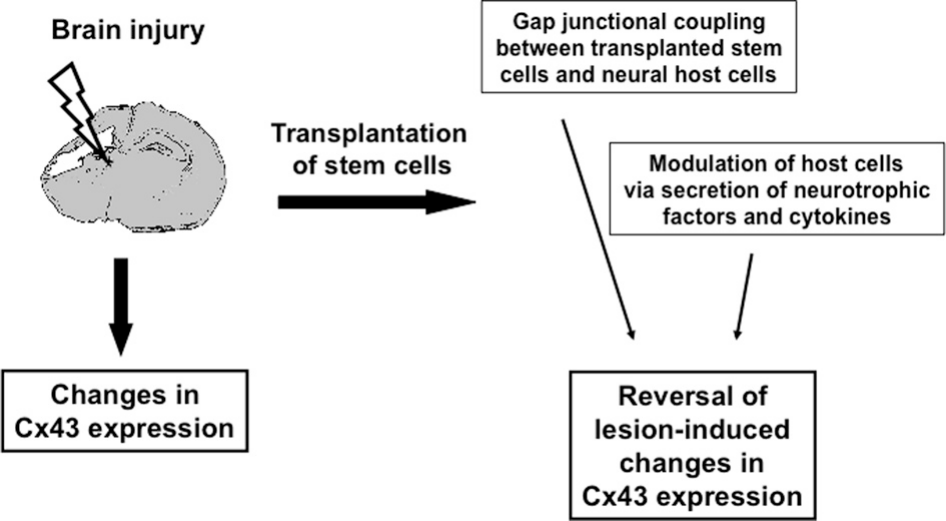
**Schematic illustration of how transplanted stem cells might modulate Cx43 changes after brain injury**.

If the beneficial effect of stem cell transplantation after brain injury was indeed related to changes in connexin expression and function, the aforementioned changes should, at least in part, be reversed in response to cell transplantation.

In the rat model of neonatal hypoxic-ischemic brain injury, a reduction of lesion-induced Cx43 expression was indeed observed as early as 1 day after transplantation of human umbilical cord blood cells. Reduced Cx43 mRNA and protein expression correlated with less astroglial activation at the structural level and with sensorimotor improvements at the functional level (Wasielewski et al., 2012). Thus, in this model the decrease of gap junction expression to almost normal levels did indeed seem beneficial for the onset of repair.

However, there are also reports on a primary reduction of Cx43 expression in response to brain injury. This observation was made in certain brain areas, for instance in blood vessels after hypoxia (Moriyama et al., 2013). In brain capillaries isolated from severely hypoxic rats, a significant reduction of Cx43 was detected. The transplantation of neural progenitor cells also caused changes in Cx43 expression in this experimental context, and the hypoxia-induced reduction was reversed in transplanted animals. Transplanted cells were shown to migrate to the affected blood vessels around the peri-infarct area (Moriyama et al., 2013).

In a model of traumatic brain injury, transplantation of neural stem cells resulted in significantly improved neurological functions in comparison with non-transplanted injured animals and these therapeutic effects were accompanied by an increase in Cx43 mRNA and protein levels (Yu et al., 2013).

In summary, beneficial effects of stem cell transplantation were accompanied by the reversal of lesion-induced changes in Cx43 expression, interestingly, irrespective of their direction. This data might point toward putative downstream regulation of gap junction expression in response to stem cell transplantation. However, speculation on mechanisms underlying the effects of transplanted cells on Cx43 expression changes is problematic for several reasons. As expression does not necessarily reflect function, the implications of the observed reduction or increase in Cx43 mRNA and protein expression require further investigation at the functional level. At this point, one has to bear in mind that, particularly for Cx43, there might be two distinct players involved in the pathophysiology, i.e., channels and hemichannels (Bennett et al., 2012). In addition, a re-distribution of Cx43 protein has been postulated to occur in response to cell damage (Hossain et al., 1994). As, in this scenario, a Cx43 pool would be utilized to re-locate the protein to different cellular compartments, changes in the total protein level, as detected by immunoblot analysis, are not to be expected. Similarly, modifications of the Cx43 protein in response to cerebral ischemia have been described: In a transient MCAO model, astrocytic Cx43 epitope masking, dephosphorylation and gap junction internalization were described (Li et al., 1998). And, even under ischemic conditions, Cx43 gap junction channels have been shown to remain functionally open *in vitro* (Cotrina et al., 1998; Li and Nagy, 2000; Contreras et al., 2002). As changes in Cx43 expression do not necessarily reflect the presence of functional channels and do not allow further discrimination of channels and hemichannels, expression data requires complementation by functional investigation.

So how might transplantation-mediated changes in Cx43 expression affect brain function and recovery of the host? On this point, several scenarios resulting in the rescue of the peri-lesion area are conceivable: An increase in cell communication between neural cells might be beneficial by leading to a faster disposal of detrimental factors or to the provision of neuroprotective substances. Alternatively, gap junctional communication might also impair healthy neighboring cells through the distribution of harmful substances. In the pathophysiological context only, i.e., without the presence of stem cells, evidence for either pathway was demonstrated *in vivo* and *in vitro* (Blanc et al., 1998; Rami et al., 2001; Frantseva et al., 2002a,b; Ozog et al., 2002; Nakase et al., 2003; Nakase and Naus, 2004). Upon application of stem cells, those studies investigating the mechanisms of their therapeutic action demonstrate (a) effects on the immune system (Rosenkranz et al., 2013; Zhang et al., 2013), (b) increased angiogenesis (Taguchi et al., 2004; Rosenkranz et al., 2012), and (c) decreased apoptosis and increased neuronal survival (Chen et al., 2003; Rosenkranz et al., 2012). However, paracrine factors might be held responsible for all of these effects. This takes us back to the capability of stem cells themselves to secrete growth factors, interleukins and chemotactic factors, which has, for instance, been demonstrated by secretome analyses of umbilical cord blood and mesenchymal stem cells (Neuhoff et al., 2007; Carvalho et al., 2011; Hsieh et al., 2013; Lavoie and Rosu-Myles, 2013; Ando et al., 2014). Examples of detected proteins include angiogenic factors, growth factors, anti-inflammatory cytokines and various chemokines (reviewed by Kupcova Skalnikova, 2013) and some of these were also detected *in vivo* (Modo et al., 2002; Vendrame et al., 2005; Yasuhara et al., 2010; Rosenkranz et al., 2012). It is feasible that these proteins of the stem cell secretome bind to neural cells in the peri-lesion area and promote neuronal and glial cell survival. For some of these factors, downstream effects such as channel or hemichannel opening have been described. This was, for instance, observed in the case of FGF-1, which was shown to open hemichannels of spinal cord astrocytes (Bennett et al., 2012). Although hypothetical, this process might provide one explanation for the relevance of gap junctions in the lesioned brain in the context of stem cell-mediated neuroprotection and repair. The causal connection between stem cell application, gap junction involvement, and brain repair, however, remains to be demonstrated *in vivo*.

In summary, these data indicate that transplantation of stem cells—independent of their source and potency—resulted in the modulation of Cx43 expression in different models of brain injury and that these changes—irrespective of the direction—are associated with the improvement of injury-induced impairments.

## Gap junctional communication between stem cells and host cells

Taking into account the expression of connexins in pluri- and multipotent stem cells themselves (Valiunas et al., 2004; Oyamada et al., 2013), recent studies indicate that gap junctional communication might even occur between transplanted cells and host cells. As investigated in a recent study analyzing Cx43 gap junctional coupling after brain damage (Jaderstad et al., 2010b), grafted murine neural stem cells formed functional gap junctions with host cells. The establishment of communicating junctions was shown to be essential for neuroprotective effects of the graft. It was postulated that homeostasis-modulating molecules were transmitted between cells, as the beneficial effect of the transplant was prevented through the inhibition of gap junctions (Jaderstad et al., 2010b). When neural stem cells were subjected to hypoxic preconditioning prior to transplantation, this effect was even enhanced (Jaderstad et al., 2010a). Another group analyzing neural stem cell transplantation in the lesioned brain identified gap junctions between implanted neural stem cells and host glial cells. They also postulated that these intercellular gap junctions might be involved in the neuroprotective and regenerative effects of transplanted neural stem cells (Talaveron et al., 2014). Interestingly, several studies report similar results in the heart following the transplantation of pluripotent stem cells (Maizels and Gepstein, 2012). The incorporation of transplanted cells into the gap junctional syncytium of the host could provide a whole new path for the transmission of neuroprotective or gliaprotective factors and prevent secondary cell death by supporting cell survival in the host tissue.

## Conclusion

The topic of stem cell-mediated changes in gap junctions is of major interest in view of the putative therapeutic potential of stem cells after brain damage. Gap junctions unquestionably modulate the outcome after brain injury, although there are contrary findings as to the course of the effects. Cx43 expression was described to be altered in different directions, and possible effects might extend from enforcement of bystander killing to the rescue of injured cells. Thus, therapeutic approaches aiming at the reduction of brain damage might include the modulation of gap junction protein expression and therefore promote neuroprotection. The number of publications describing changes in connexin expression upon transplantation of stem cells is still limited. In our view, changes in gap junctional communication provides one plausible explanation for the beneficial effects observed upon stem cell transplantation—the distribution of neuroprotective factors might be enhanced in the peri-lesional regions of the brain through increased intercellular communication between host cells, and possibly even between host and transplanted cells.

### Conflict of interest statement

The authors declare that the research was conducted in the absence of any commercial or financial relationships that could be construed as a potential conflict of interest.
